# 1459. Prevalence of Enterovirus and Parechovirus in Children with Acute Gastroenteritis and in Healthy Controls over a 7-year Period; 2011-2018

**DOI:** 10.1093/ofid/ofac492.1286

**Published:** 2022-12-15

**Authors:** Anjana Sasidharan, Kayla Shore, Brian R Lee, Christopher J Harrison, Jennifer E Schuster, Mary E Moffatt, Kirsten Weltmer, Mary Wikswo, Brian D Emery, Steven Oberste, Rangaraj Selvarangan

**Affiliations:** Childrens Mercy Hospital, Missouri, Kansas; Children's Mercy Hospital Kansas City, Kansas City, Missouri; Children's Mercy Kansas City, Kansas City, Missouri; Children's Mercy - Kansas City, Kansas City, Missouri; Children's Mercy Kansas City, Kansas City, Missouri; Children's Mercy Kansas City, University of Missouri Kansas City School of Medicine, Kansas City, Missouri; Children's Mercy Kansas City, Kansas City, Missouri; Centers for Disease Control and Prevention, Atlanta, Georgia; Centers for Disease Control and Prevention, Atlanta, Georgia; CDC, Atlanta, Georgia; Children's Mercy, Leawood, Kansas

## Abstract

**Background:**

As of 2017 (WHO, 2017), diarrheal disease ranked second as a cause of worldwide mortality for children under five years of age. Approximately 50-70% of acute gastroenteritis (AGE) is viral in etiology, with commonly detected viruses including norovirus, rotavirus, and adenovirus. However, the epidemiology of less commonly detected viruses, specifically enterovirus (EV) and parechovirus A (PeV-A), associated AGE in the United States is not well described. The purpose of our study was to determine the prevalence of EV and PeV-A in children with AGE vs. healthy controls (HC) over a 7-year period.

**Methods:**

From December 2011 – November 2018, we screened stool samples from children less than 18 years of age prospectively enrolled in Children’s Mercy-Kansas City’s (CM-KC) site for the CDC’s New Vaccine Surveillance Network; 3005 samples from subjects presenting with AGE and 1097 from HC. Samples from 2011 – 2016 (AGE: 2453; HC:752) and 2017 – 2018 (AGE:552; HC:344) were tested at CDC and CM-KC respectively by a real-time-PCR assay using specific EV and PeV-A primers targeting the highly conserved 5’ untranslated region. Demographic data were collected from EMR.

**Results:**

Among 3005 AGE samples, EV was detected in 12.5% (n=386/3004), and PeV-A in 10.3% (n=252/3005). Among 1097 HC samples, EV was detected in 9.0% (99/1096), and PeV-A in 11.9% (130/1097). In 2014-2015 EV detection in AGE was highest (17.9%) among all years and significantly higher (p=0.004) than in HC samples (9.1%), whereas PeV-A detection in AGE was 9.5% vs. 15.6% in HC samples, p=0.008 (Table 1). Co-infections with EV and PeV-A were seen in 55 AGE and 21 HC. Most EV detections (45.1%) were in 1- to 2-year-olds, whereas PeV-A detections (47.3%) were in children < 1 year old (Table 2). Both EV (58.3%) and PeV-A (48.4%) detections were significantly more frequent in male children (p=0.006). The highest frequency of EV detections was in summer to fall months, and for PeV-A in late summer through early winter.

**Figure:**
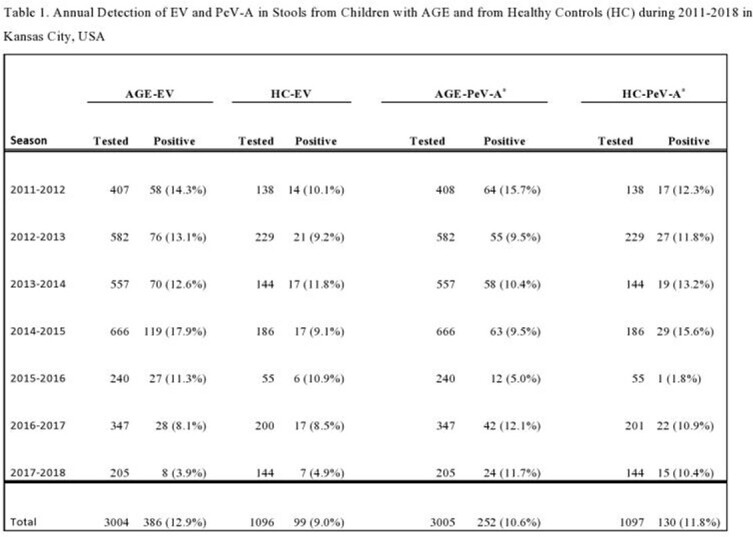

**Figure:**
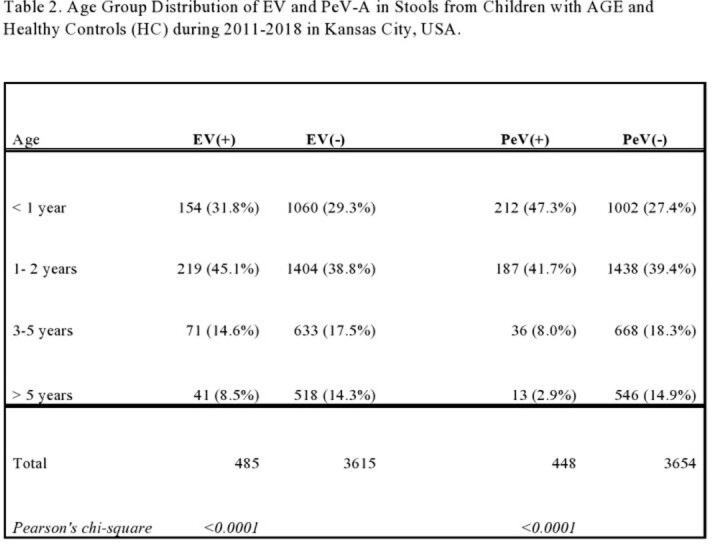

**Conclusion:**

We report a higher prevalence of EV infections in AGE, and PeV-A infections in HC during 2011 to 2018, plus their seasonal and age distributions. Although our data do not currently demonstrate an association between detection of EV or PeV-A types and AGE, additional data could provide more clarity into a potential association.

**Disclosures:**

**Brian R. Lee, PhD, MPH**, CDC: Grant/Research Support|Merck: Grant/Research Support **Christopher J Harrison, MD**, Astellas: Grant/Research Support|GSK: Grant/Research Support|Merck: Grant/Research Support|Pediatric news: Honoraria|Pfizer: Grant/Research Support **Mary E. Moffatt, M.D.**, Becton and Dickinson and Company: Stocks/Bonds|Biogen: Stocks/Bonds|Coloplast B: Stocks/Bonds|Express Scripts: Stocks/Bonds|Novo Nordisk A/S Spons ADR: Stocks/Bonds|Novo Nordisk A/S-B: Stocks/Bonds|Steris PLC: Stocks/Bonds|Stryker Corp: Stocks/Bonds|Thermo Fisher Scientific: Stocks/Bonds **Rangaraj Selvarangan, BVSc, PhD, D(ABMM), FIDSA, F(AAM)**, BioFire: Grant/Research Support|Luminex: Grant/Research Support.

